# Tolerability and impact on postoperative morbidity of preoperative bowel preparation in Crohn’s disease patients: results of prospective observational study

**DOI:** 10.1007/s00384-026-05084-z

**Published:** 2026-02-02

**Authors:** Igors Iesalnieks, Aline Schmitz, Nils Hinrichs, Dominika Ivanecka, Zdenek Kala, Tomas Grolich, Lumir Kunovsky

**Affiliations:** 1 Department of Surgery, Evangelisches Krankenhaus Kalk, Buchforststr. 2, 51103 Cologne, Germany; 2 Institute of Artificial Intelligence in Medicine, Deutsches Herzzentrum Der Charité, Berlin, Germany; 3 Department of Surgery, University Hospital Brno, Faculty of Medicine, Masaryk University, Brno, Czech Republic; 4 2nd Department of Internal Medicine - Gastroenterology and Geriatrics, University Hospital Olomouc, Faculty of Medicine and Dentistry, Palacky University Olomouc, Olomouc, Czech Republic; 5 Department of Gastroenterology and Digestive Endoscopy, Masaryk Memorial Cancer Institute, Brno, Czech Republic

**Keywords:** Bowel resection, Crohn’s disease, Mechanical bowel preparation, Oral antibiotics, Posoperative complications, Surgery

## Abstract

**Background:**

Preoperative mechanical bowel preparation (MBP) and oral antibiotics (OA) are widely used to decrease the risk of postoperative septic complications after colorectal resections. Unfortunately, it is not clear whether bowel preparation can lead to symptoms of small bowel obstruction, which might even increase the risk of postoperative morbidity.

**Methods:**

Consecutive Crohn’s disease patients undergoing bowel resections with formation of ileocolic or colocolic anastomosis were included in the present prospective observational study. Urgent surgery, surgery without preoperative MBP, colorectal cancer, and fecal diversion were exclusion criteria. A polyethylene glycol solution (2 L) was used for MBP. OA consisted of paramomycin and metronidazole taken at 7 p.m. and 11 p.m. at the evening before surgery. Occurrence of complications at the anastomotic site (leakage, peritonitis, abscess, or fistula in direct proximity to the anastomosis) was a primary outcome measure. Complications of MBP were recorded. Mechanical bowel preparation was defined as “incomplete” when patients took a lesser amount of MBP solution than scheduled.

**Results:**

Between 2016 and 2024, ileocolic or colorectal resections with formation of an anastomosis were performed in 284 patients with Crohn’s disease. Nausea, vomiting, or abdominal pain occurred during the MBP in 29% of patients (*n* = 78), leading to termination of intake in 53 patients (19%)**.** Women (*p* < 0.001), patients hospitalized urgently because of acute abdominal pain (*p* = 0.008), patients presenting with severe anemia before surgery (*p* = 0.007), and patients scheduled for resections completed by ileocolic anastomosis as opposed to colocolic or colorectal anastomosis (*p* = 0.01) demonstrated a significantly increased risk of incomplete MBP. Thirty-two percent of patients demonstrated apparent dilatation of small bowel at the time of surgery. The incidence of anastomotic complications was 4% in patients who were able to complete MBP and 7.5% after an incomplete MBP (*p* = 0.27). There were no deaths. The conversion rate from laparoscopy to open surgery was increased in patients with small bowel dilatation (17% vs. 6%); however, the difference was not statistically significant (*p* = 0.13).

**Conclusion:**

There is a considerable incidence of obstructive symptoms after preoperative mechanical bowel preparation in Crohn’s disease patients. Nevertheless, an incomplete MBP is not associated with increased risk of intra- or postoperative complications and can be used safely in that particular population.

**Supplementary Information:**

The online version contains supplementary material available at 10.1007/s00384-026-05084-z.

## Introduction

Preoperative mechanical bowel preparation in combination with oral antibiotics (MBP + OA) has been demonstrated to reduce the risk of postoperative surgical site infections and anastomotic leaks in patients undergoing colorectal resections [1, 2]. However, the vast majority of patients included in studies on preoperative bowel preparation undergo surgery for cancer. Data on the impact of MBP + OA on postoperative morbidity in patients with Crohn’s disease are very scarce [3]. Moreover, the readiness to apply MBP in Crohn’s disease patients is limited due to a high prevalence of obstructive symptoms in that particular population. Acute obstructive symptoms caused by MBP in the night before surgery might lead to serious fluid and electrolyte disturbances and even increase the risk of postoperative intraabdominal complications.

In a large retrospective analysis, MBP (with or without OA) has been shown to significantly decrease the risk of postoperative intraabdominal septic complications in Crohn’s disease patients [3]. However, 15% of patients in that study developed side effects—mostly vomiting and abdominal pain. Patients who discontinued MBP due to side effects had a statistically non-significantly increased risk of postoperative morbidity. Unfortunately, that study had several limitations: its retrospective nature might have led to underestimation and underreporting of MBP-induced complications. Also, the majority of patients did not receive oral antibiotics, which might have been the reason for the overall high complication rate even in patients in the MBP group [3].

The present prospective observational study was conducted to obtain exact data on side effects and discontinuation rates of preoperative MBP in patients undergoing bowel resection for Crohn’s disease. Also, the impact of the MBP discontinuation on postoperative morbidity was analyzed. The primary endpoint of the study was the incidence of postoperative anastomotic complications.

## Methods

Consecutive patients with Crohn’s disease undergoing elective ileocolic or colorectal resections completed by an anastomosis were included in the present prospective observational study.

### Preoperative bowel preparation

The MBP was started between 1 p.m. and 3 p.m. at the day before surgery. According to the producer’s package insert, MBP consisted of 2 L of polyethylene glycol (PEG) bowel lavage solution (“Moviprep” by Fa. Norgine, Marburg, Germany). Oral antibiotics, which included Metronidazole 1 g and Paromomycine 2 g, were given at 7 p.m. and the same dose at 11 p.m. The intake of the MBP solution had to be completed before starting oral antibiotics; however, patients were allowed to drink the MBP solution also thereafter in case when abdominal pain, nausea, or vomiting precluded faster intake. Perioperative intravenous antibiotics (Metronidazole and a third-generation Cephalosporine) were given 30 to 60 min before surgery.

### Exclusion criteria

1. Resections completed with any kind of ostomy formation
2. Mere strictureplasties
3. Ostomy closures
4. Emergency surgeries
5. Patients with mere small bowel anastomosis
6. Patients with colorectal malignancy

Patients who underwent emergency surgery were excluded because they were not able to take MBP in a short time span between emergent hospitalization and surgery. In contrast, MBP was given to patients who were hospitalized emergently but whose condition improved as far as to enable them to undergo elective resection. Thus, latter patients have been included in the current study.

### Data collection

At the morning before the surgery, all patients were asked whether they had had any side effects following MBP intake and whether they were able to drink the total amount of MBP solution they were scheduled for. An “incomplete MBP” was defined as intake of less than scheduled 2 L of MBP solution. “Preoperative optimization” was defined as at least 7 days of preoperative nutritional treatment and/or abscess drainage and/or intravenous antibiotic treatment and/or steroid tapering. “Anastomotic complications” were defined as the occurrence of postoperative anastomotic leak, abscess or peritonitis close to the anastomosis. “Postoperative intraabdominal septic complications” included anastomotic complications as well as any other kind of intraabdominal abscess, small bowel fistula, and peritonitis. Preoperative hemoglobin level of < 10 g/dl was defined as “severe anemia”. Anastomotic complications and postoperative intraabdominal septic complications were diagnosed by CT scan and/or repeat laparotomy.

The *primary endpoint* of the study was the occurrence of postoperative anastomotic complications. It was hypothesized that patients who were not able to complete MBP are at a significantly increased risk of postoperative anastomotic complications.

The *secondary endpoint* of the study was the occurrence of postoperative intra-abdominal septic complications.

### Statistics

The sample size calculation was based on the expected anastomotic complication rate of 1.5% in patients with complete and of 10% in patients with incomplete MBP [4]. The incidence of incomplete MBP was expected to be 20% [4]. To reach a power of 80%, it was estimated that a total of 275 patients would be required to detect the difference of anastomotic complications between 10 and 1.5% with a type I error of 0.05.

The Wilcox rank sum test was used in order to compare groups regarding continuous variables. The *χ*^2^ test was applied for all categorical variables. All *p* values were 2-sided. The significance level was set at 0.05. Variables that were found to be significantly associated with the primary or secondary outcome or incomplete MBP in the univariate analysis were included in a subsequent multivariate analysis using logistic regression.

The study has been approved by the local ethical committee. The study adhered to the Strengthening the Reporting of Observational Studies in Epidemiology (STROBE) recommendations (see supplementary material).

## Results

### Baseline characteristics

284 consecutive patients with Crohn’s disease undergoing elective ileocolic or colorectal resections concluded with a formation of an ileocolic, colocolic, or colorectal anastomosis between 2016 and 2024 were included in the present prospective observational study. Patients’ demographics and preoperative characteristics are summarized in Table 1. 35 patients (12%) admitted to the hospital through the emergency department due to acute abdominal pain. They underwent surgery after they absolved a median of 10 days of preoperative optimization.

**Table 1:** Preoperative demographic and disease characteristics

Variable	N (%)
Sex	
Male	134 (47%)
Female	150 (53%)
Age, mean (range)	41.4 years (17–78)
Disease localization	
L1—ileum	220 (79%)
L2—colon	16 (6%)
L3—ileum + colon	41 (15%)
Disease behavior	
B1—non stricturing/non-penetrating	16 (6%)
B2—stricturing	132 (47%)
B3—penetrating	136 (47%)
Previous bowel resections	85 (30%)
Nicotine smoking	102 (38%)
Preoperative medication	
No immunosuppression/no biologics/no biologicals	111 (40%)
Steroids	45 (16%)
Azathioprine	39 (14%)
Methotrexate	5 (2%)
Infliximab/Adalimumab	67 (24%)
Vedolizumab	12 (4%)
Ustekinumab	37 (13%)
Other biologicals	4 (1%)
Preoperative weight loss > 5% of body mass	102 (37%)
Preoperative severe anemia*	14 (5%)
Preoperative optimization**	56 (20%)

^*^Hemoglobin level < 10 g/dl

^**^Consisting of preoperative nutritional support, antibiotics, percutaneous abscess drainage, steroid tapering—for 7 to 14 days

245 patients underwent ileocolic resections (86%), 30 patients underwent colonic resections (10.5%), and 9 underwent both types of resections during the same procedure (3.5%). Laparoscopic resections were performed in 227 patients (80%), open in 34 patients (12%), and the surgery was converted from laparoscopic to open in the remaining 23 patients (8%). Out of 39 colonic resections, 8 were right colectomies, 10 were transversal colon resections, 21 were left-sided colectomies.

### Postoperative morbidity

Postoperative complications occurred after 44 surgeries (15.5%); no patient died. The incidence of intraabdominal septic complications was 7% (*n* = 19), and complications at the anastomosis occurred in 13 patients (5%). Only 5 patients (1.8%) had evident anastomotic leaks—three at the ileocolic and two at colocolic anastomoses. Five patients developed peritonitis or abscesses at the ileocolic anastomoses without apparent disruption of the anastomosis. Three more patients, after ileocolic resections, were readmitted to hospital several weeks after an uneventful postoperative course, with leakages at the closed ileum stump of the side-to-side anastomosis. Table 2 lists all intraabdominal septic complications.

**Table 2:** List of postoperative intraabdominal septic complications

Type of complications	Number
Anastomotic complications	
Apparent anastomotic leak*	5
Abscess at the anastomosis	1
Peritonitis at the anastomosis	4
Leakage at the closed ileum stump of the side-to-side ileocolic anastomosis	3
Non-anastomotic complications	
Small bowel perforation	2
Sigmoid perforation	1
Diffuse peritonitis	2
Mesenteric abscess	1

^*^Visible disruption of anastomotic line at the time of revision surgery, at endoscopy or as evidenced by contrast extravasation at abdominal CT scan or fecal discharge from an intraabdominal drainage

### MBP tolerability

All patients underwent preoperative mechanical bowel preparation (MBP). Two patients refused to take oral antibiotics. Complications of MBP occurred in 78 patients (29%)—vomiting (*n* = 38), nausea (*n* = 43), and abdominal pain (*n* = 11). Fifty-three patients (19%) were not able to complete MBP mostly due to complications, 12 of which drank less than a half of MBP solution. Female sex, emergency hospitalization, decreased hemoglobin level, and scheduled ileocolic resection were associated with an increased risk of incomplete MBP (Table 3). All patients undergoing a mere colorectal resection without an additional ileocolic resection were able to complete the MBP. Intraoperatively, significant small bowel dilatation was documented in 32% (30 of 93 patients with available data) of patients. The conversion rate from laparoscopic to open surgery was higher in patients with small bowel dilatation (17%) than in patients without small bowel dilatation (6%); however, the difference was not statistically significant (*p* = 0.13). There was no statistically significant difference between the first five years (2016–2020) and the last four years (2021–2024) of the study regarding the proportion of patients unable to complete MBP (16% vs. 21%, *p* = 0.36).

**Table 3:** Factors associated with incomplete preoperative MBP*

	Complete MBP	Incomplete MBP	*p*
	*N* = 230 (%)	*N* = 53 (%)	
Age, mean	41.9 years	39.0 years	0.219
Female sex	111 (48)	39 (74)	0.001
Preoperative hemoglobin level, g/dl	13.20	12.70	0.007
Preoperative weight loss	81 (36)	20 (39)	0.822
Smokers	77 (35)	25 ( 51)	0.051
Emergency hospitalization	22 (10)	13 (25)	0.008
Previous bowel resections	73 (32)	12 ( 23)	0.256
Preoperative steroid intake	35 (16)	10 ( 19)	0.693
Anti -TNF treatment	55 (24)	12 ( 23)	0.986
Ustekinumab treatment	25 (11)	12 ( 23)	0.039
Preoperative abscess	32 (28)	9 ( 41)	0.343
Clinical behavior			0.398
Non-stricturing/non-penetrating	14 ( 6)	2 (4)	
Stricturing	103 (45)	29 ( 55)	
Penetrating	113 (49)	22 ( 42)	
Use of preoperative optimization	41 (18)	15 ( 28)	0.125
Scheduled ileocolic resection	199 (87)	53 (100)	0.010
Scheduled colonic resection	37 (16)	2 (4)**	0.034

*Missing data on completeness of MBP in one patient

**Additionally to an ileocolic resection

### Study endpoints

Patients having an incomplete MBP did not demonstrate an increased rate of postoperative anastomotic complications, nor did they develop more intraabdominal septic complications (Table 4). The multivariate analysis included the variables found to be associated with the primary or secondary outcome in a univariate analysis. Only microscopically positive resection margin was associated with an increased risk of anastomotic complications (OR 3.74; 95% CI: 1.08–12.9; *p* = 0.037), and the preoperative weight loss of at least 5% of body mass was associated with postoperative intraabdominal septic complications (OR 3.20; 95% CI: 1.09–9.41; *p* = 0.034).

**Table 4:** Univariate analysis of risk factors associated with postoperative anastomotic and all intraabdominal septic complications

Variable	Anastomotic complications *N* (%)	Intraabdominal septic complications, *N* (%)
Sex		
F	6/149 (4%)	8/149 (5%)
M	7/135 (5%)	11/135 (8%)
Age		
≤ 40 years	8/155 (5%)	9/155 (6%)
> 40 years	5/129 (4%)	10/129 (8%)
Nicotine smoking		
Yes	7/102 (7%)	10/102 (10%)
No	6/170 (4%)	9/170 (5%)
Weight loss > 5% of body mass		
Yes	7/102 (7%)	13/102 (13%)*
No	6/173 (4%)	6/173 (4%)
Emergent hospitalization		
Yes	3/35 (9%)	6/35 (17%)*
No	10/247 (4%)	13/247 (5%)
Preoperative abscess		
Yes	3/41 (7%)	6/41 (15%)
No	10/243 (4%)	13/243 (5%)
Penetrating disease		
Yes	6/136 (4%)	11/136 (8%)
No	7/148 (5%)	8/148 (5%)
Extraintestinal disease manifestations		
Yes	7/121 (6%)	10/121 (8%)
No	4/148 (3%)	6/148 (4%)
Severe preoperative anemia (Hb < 10 g/dl)		
Yes	2/14 (14%)	4/14 (29%)*
no	11/266 (4%)	15/266 (6%)
Steroid intake		
Yes	1/45 (2%)	2/45 (4)
No	12/235 (5%)	17/235 (7%)
Azathioprine intake		
Yes	0/39 (0)	1/39 (3%)
No	13/245 (5%)	18/245 (7%)
Anti-TNF treatment		
Yes	2/67 (3%)	4/67 (6%)
No	11/217 (5%)	15/217 (7%)
Ustekinumab treatment		
Yes	4/37 (11%)	4/37 (11%)
No	9/247 (4%)	15/247 (6%)
Previous bowel resection		
Yes	4/85 (5%)	4/84 (5%)
No	9/199 (5%)	15/199 (8%)
Preoperative optimization		
Yes	2/56 (4%)	6/56 (11%)
No	11/228 (5%)	13/228 (6%)
**Incomplete mechanical bowel preparation**		
**Yes**	**4/53 (7.5%)**	**5/53 (9%)**
**No**	**9/230 (4%)**	**14/230 (6%)**
**Complications of MBP**		
**Yes**	**4/78 (5%)**	**6/78 (8%)**
**No**	**7/189 (4%)**	**10/189 (5%)**
Ileocolic resection		
Yes	12/254 (5%)	16/254 (6%)
No	1/30 (3%)	3/30 (10%)
Laparoscopic resection		
Yes	10/250 (4%)	15/250 (6%)
No (open or converted)	3/34 (9%)	4/34 (12%)
Microscopically positive resection margins		
Yes	6/59 (10%)*	7/59 (12%)*
No	4/178 (2%)	7/178 (4%)
Time period		
2016–2020	6/118 (5%)	9/118 (8%)
2021–2024	7/166 (4%)	10/166 (10%)

^*^p < 0.05

### Subgroup analysis: previous bowel resections

For 60 (21%) patients, the current bowel resection was their second; it was the third resection in 22 cases (8%), the fourth in 2 patients, and the fifth in 1 patient. Patients with previous resections were significantly older (49 years vs. 38 years, *p* < 0.001), they were more often smokers (47% vs. 33%, *p* = 0.041), they presented more frequently with extraintestinal symptoms (56% vs. 40%, *p* = 0.017), and enterocutaneous fistulae (6% vs. 0.5%, *p* = 0.010). Also, they underwent less laparoscopic surgeries (69% vs. 96%, *p* < 0.001), and their resected specimens were shorter (16 cm vs. 24 cm, *p* < 0.001). However, neither the risk to develop MBP-induced side effects nor the postoperative morbidity differed between patients undergoing their first or repeat resections (Tables 3 and 4).

## Discussion

Current study demonstrated a considerable incidence of side effects caused by preoperative MBP (29%) in Crohn’s disease patients. 19% of patients were not able to complete MBP, and about one third demonstrated small bowel dilatation at the time of surgery. Nevertheless, the occurrence of side effects which led to premature termination of MBP did not lead to an increased postoperative morbidity.

Since late seventies [1, 5], preoperative MBP combined with the intake of OA has been shown to decrease the risk of postoperative anastomotic leak and surgical site infections (SSI) after colorectal surgery. During the last four decades, however, the controversy has amounted. Specifically, the widespread introduction of enhanced recovery after surgery (ERAS) programs across the world questioned the necessity of MBP. Several European studies did not demonstrate any advantages of preoperative MBP with or without oral antibiotics [6–8] which led to a widespread elimination of MBP from preoperative routine, especially in Europe. However, the controversy increased after the results of a prospective randomized study by Bretagnol et al. were published in 2010, which demonstrated significantly increased morbidity after rectal cancer surgery if preoperative MBP was skipped [9]. In 2015, three large population-based studies from the USA demonstrated a decreased risk of anastomotic leak and/or SSI if MBP + OA was used as compared to no bowel preparation, to MBP alone, or to OA alone [10–12]. Finally, the two most recent prospective randomized studies from Finland failed to demonstrate any protective effect of MBP + OA in patients undergoing colonic resection [13] but they showed a significantly decreased risk of septic complications after rectal resection in patients receiving MBP + OA as compared to MBP alone in a non-IBD population [2]. As for now, preoperative MBP + OA is recommended by the American Society of Enhanced Recovery [14] in patients undergoing colorectal resections. However, the European ERAS society [15] recommends to consider MBP with OA only before rectal resection.

Unfortunately, IBD patients were mostly not included in any of the abovementioned studies, or their proportion was too low to perform any subgroup analysis. Although preoperative optimization is strongly recommended prior to bowel resection for Crohn’s disease by numerous guidelines and meta-analyses [16], preoperative bowel preparation is almost never discussed. As for now, there are only a few studies on preoperative bowel preparation in Crohn’s disease patients. Iesalnieks et al. [3] demonstrated in 2018 a significant decrease in the intraabdominal septic complication rate after MBP (without OA) in a large retrospective analysis. In a retrospective study by Unger et al. [17], Crohn’s disease patients undergoing colorectal resections benefited from MBP (without OA) as well; however, it was not the case for patients undergoing ileocolic or small bowel resections in particular. Furthermore, the risk of anastomotic leak was decreased (Odd ratio 0.73) by preoperative MBP + OA in Crohn’s disease patients in a global cohort analysis using the NSQIP database, which included 6244 patients [18]. Finally, the incidence of incisional SSI was decreased in Crohn’s disease patients undergoing intestinal resections after MBP + OA as compared to MBP alone in a prospective randomized study by Uchino [19]. However, there was no difference in the occurrence of organ space infections between groups in the latter study.

The reluctance to apply MBP (with or without OA) to Crohn’s disease patients is mainly caused by the fear of obstructive symptoms which might be provoked by MBP, especially in the presence of stricturing disease. Postoperative morbidity could even be increased if acute symptoms of ileus occurred at the night before surgery. Indeed, patients who were not able to complete the preoperative MBP were characterized by several distinctive features: they presented more often with acute obstructive symptoms which were caused by severe ileocolonic disease necessitating emergency hospitalization. Thus, apparently, there is some correlation between the severity of obstructive symptoms before surgery and the incidence of obstructive symptoms after the intake of MBP solution. In contrast, all patients with mere colonic disease scheduled for a colorectal resection were able to complete MBP. Nevertheless, after adjustment for these confounders, the risk of postoperative anastomotic leak and intraabdominal septic complications was not increased neither in patients with side-effects of MBP nor it was increased after an incomplete MBP. Thus, the use of MBP can be viewed as safe, although the incidence of side-effects is considerable.

The colon cleansing is inadequate in 18 to 33% of patients undergoing elective colonoscopy after MBP [20]. The PEG solution is generally used with a large volume (2 to 4 L) prior to colonoscopy to achieve adequate cleansing. However, the large volume of MBP solution and the bad taste of PEG are both associated with abdominal pain and vomiting in both non-IBD and IBD patients, even in the presence of quiescent disease. Non-PEG solutions are not recommended for elective colonoscopy in IBD patients since they are believed to cause mucosal inflammation [21]. However, since they are used as low-volume solutions and are more palatable than the PEG solution, they might be a better choice in the preoperative setting. Also, the results of the present study suggest that MBP + OA might be effective even at a lower MBP volume than 2 L. We chose those 2 L according to manufacturers’ recommendations. However, those recommendations are aimed to achieve certain degree of cleansing preparing for a colonoscopy. As for now, it is not known whether the degree of preoperative colonic cleansing is directly linked to a lower complication rate. Our study at least suggests that that is not the case. In fact, a lower volume of MBP could lead to less side effects and better patients’ adherence. Before the results of the present study, patients were recommended to try their best to take the full amount of the scheduled PEG solution. However, from now on, we recommend stopping the intake of MBP solution as soon as the first signs of obstruction occur.

The present study was not conducted in order to prove the efficacy of preoperative MBP + OA in Crohn’s disease patients. The main aim was to evaluate if there was any harm in those patients who developed obstructive symptoms following the intake of MBP solution, which was apparently not the case. Nevertheless, there is still a question: should we accept an additional suffering (i.e., side effects) caused by preoperative MBP in order to prevent even more suffering (i.e., postoperative morbidity)? The current study was not sufficient to address that particular question. An anastomotic leak occurred in 1.8% of patients; altogether, 5% had septic complications in direct proximity to the anastomosis, and 7% had intraabdominal septic complications. Those results match the postoperative morbidity documented in many recent studies on bowel resections in Crohn’s disease. Unfortunately, most authors do not mention whether MBP has been used or not in their cohorts. However, many surgeons seem not to apply MBP in ileocolic resections. Nevertheless, there is an urgent need for a prospective randomized study comparing MBP + OA to no MBP at all or comparing MBP + OA to OA alone in Crohn’s disease patients. Discussions and recommendations regarding preoperative bowel preparation in IBD patients should be included in current reviews and guidelines.

### Limitations of the study

The study was possibly underpowered, as the significantly higher incidence of postoperative anastomotic complications was expected in patients who were not able to complete MBP [4]. Since there was a significant difference in MBP tolerability (and incidence of incomplete MBP) between patients undergoing ileocolic as opposed to other colorectal resections, focusing on ileocolic resections would probably sharpen the study profile. Despite the prospective nature of the study, some variables (smoking, extraintestinal manifestations, intraoperative bowel dilatation) were incomplete. Also, “Bowel dilatation” was not standardized. The proportion of laparoscopic surgery increased during the whole study period, possibly fostering changing morbidity patterns over the study period.

## Significance statement

Preoperative bowel preparation has been discussed to be a major factor in the prevention of postoperative morbidity in patients undergoing colorectal resections. However, there is a significant lack of data regarding the feasibility, impact, and morbidity of mechanical bowel preparation in Crohn’s disease patients. The present study conducted in a large prospective patients’ collective provides valuable and reliable data clarifying the role of preoperative bowel preparation in that particular population.

## Supplementary Information

Below is the link to the electronic supplementary material.

ESM 1 (DOCX 18.1 KB)

## Data Availability

The data that support the findings of this study are available from the corresponding author, [I.I.], upon reasonable request.
