# Supplementary material for: Tolerability and impact on postoperative morbidity of preoperative bowel preparation in Crohn’s disease patients: results of prospective observational study
Source: Int J Colorectal Dis. 2026 Feb 2;41(1):53. doi: 10.1007/s00384-026-05084-z (PMC12864251; doi:10.1007/s00384-026-05084-z)
Supplement: Supplementary file 1 — (DOCX 18.1 KB) [file 384_2026_5084_MOESM1_ESM.docx]

Supplementary material 1.

Checklist according to STROBE recommendations

| 1. Title and abstract | Study`s design was included in the title. The abstract is informative and gives a comprehensive insight in what was done and what was found |
| --- | --- |
| 2-3.Background and objectives | There is a lack of reliable data on safety of MBP in Crohn`s disease patients. The aim oft the present study was to close that gap and to analyze the impact of side-effects caused by MBP on postoperative morbidity |
| 4. Study design | The observational nature oft he study was emphasized at the beginning oft he METHODS section |
| 5. Setting | Inclusion criteria, study period and methods of data collection were demonstrated clearly in METHODS section |
| 6. Participants | All consecutive Crohn`s disease patients undergoing ileocolic or colocrectal resection with an anastomosis were included. |
| 7. Variables | Outcome parameters (anastomotic complications) and potential confounders other than incomplete MBP were listed in Table 3 |
| 8. Data measurments | Statistical methods were clearly explained |
| 9. Bias | There was no apparent bias in the present study. |
| 10. Study size | The sample size calculation was included in METHODS section |
| 11. Quantitave variables | The handling of quantitative variables was described in METHODS section |
| 12. Statistical methods | The study statistics were managed by professional statistician (N.H.) and explained in METHODS section |
| 13. Participants | The number of participitants did not change throughout the time oft he study |
| 14. Descriptive data | Characteristics of study participants and missing data are clearly demonstrated in Table 1 |
| 15. Outcome data | All numbers of Outcome events have been demonstrated |
| 16. Main results | Only age was dichotimized according to its median value |
| 17. Other analyses | No |
| 18. Key results | Key results have been summarized at the beginning of the DISCUSSION |
| 19. Limitations | Limitations of the study are mentioned in the last section |
| 20. Interpretation | Interpretation of study results are provided in the last section of the manuscript |
| 21. Generalisability | Generalisability was also discussed in the last section |
| 22. Funding | No Funding was used |
